# Maternal body mass index and risk of neonatal adverse outcomes in China: a systematic review and meta-analysis

**DOI:** 10.1186/s12884-019-2249-z

**Published:** 2019-03-29

**Authors:** Lei Liu, Yanan Ma, Ningning Wang, Wenjing Lin, Yang Liu, Deliang Wen

**Affiliations:** 1 School of Public Health, Dalian Medical University, Dalian, Liaoning Province 116044 People’s Republic of China; 2 School of Public Health, China Medical University, No.77 Puhe Road, Shenyang North New Area, Shenyang, Liaoning Province 110122 People’s Republic of China

**Keywords:** Maternal BMI, Meta-analysis, Cohort study, Neonatal outcomes

## Abstract

**Background:**

Maternal body mass index is linked to short- and long-term unfavorable health outcomes both for child and mother. We conducted a systematic review and meta-analysis of population-based cohort studies to evaluate maternal BMI and the risk of harmful neonatal outcomes in China.

**Methods:**

Six databases identified 2454 articles; 46 met the inclusion criteria for this study. The dichotomous data on maternal BMI and harmful neonatal outcomes were extracted. Pooled statistics (odds ratios, ORs) were derived from Stata/SE, ver. 12.0. Sensitivity analyses assessed the robustness of the results. Meta-regression and subgroup meta-analyses explored heterogeneity.

**Results:**

The meta-analysis revealed that compared with normal BMI, high maternal BMI is associated with fetal overgrowth, defined as macrosomia ≥4000 g (*OR* 1.91, 95% CI 1.75–2.09); birth weight ≥ 90% for gestational age (*OR* 1.88, 95% CI 1.64–2.15); and increased risk of premature birth (*OR* 1.38, 95% CI 1.25–2.52) and neonatal asphyxia (*OR* 1.74, 95% CI 1.39–2.17). Maternal underweight increased the risk of low birth weight (*OR* 1.61, 95% CI 1.33–1.93) and small for gestational age (*OR* 1.75, 95% CI 1.51–2.02).

**Conclusions:**

Raised as well as low pre-pregnancy BMI is associated with adverse neonatal outcomes. Management of weight during pregnancy might help reduce their adverse neonatal outcomes in future intervention studies or programmes.

**Electronic supplementary material:**

The online version of this article (10.1186/s12884-019-2249-z) contains supplementary material, which is available to authorized users.

## Background

Obesity is a global health problem [1]. Among adults of all ages, women generally have higher rates of obesity than men [2]. Rates of obesity in pregnancy are increasing, particularly in developed countries [3, 4]. In the USA, a survey indicated that 55.8% of women of childbearing age (20–39 years) were overweight or obese, defined as having a BMI of 25 or higher [5]. It is worth emphasizing that China is the country with the highest rate of childhood obesity [2]. Therefore, overweight and obesity is an increasing health burden in China. The rate of maternal overweight and obesity is 25.1% as reported in the Chinese population [6]. There is considerable evidence that maternal obesity during gestation increases the incidence of complications such as childhood obesity, diabetes, cardiovascular diseases, several types of cancer, and metabolic syndrome at multiple life stages in the offspring [4]. In contrast, maternal underweight has a protective effect on these pregnancy complications except for the slightly increased risks of having a baby with low birth weight and intrauterine growth restriction. As many of the physiological changes of pregnancy associated with maternal obesity are present from early pregnancy onward, reducing maternal obesity before conception is probably the best strategy to decrease the health burden of adverse fetal and birth outcomes [7].

Several observational studies and systematic reviews have provided a connection between maternal BMI during pre-pregnancy or early gestation and adverse perinatal outcomes [8]. Infants of overweight or obese mothers are affected by various pregnancy comorbidities including gestational diabetes, gestational hypertension, preeclampsia, premature birth (PTB), macrosomia and stillbirth [9, 10]. Pre-pregnancy underweight remains a significant health problem and is associated with low birth weight (LBW) and small for gestational age (SGA) [11, 12]. However, not all studies show a statistically significant relationship and no comprehensive appraisals of these outcomes with well-designed cohort studies have been conducted in China.

China has made impressive achievements in improving maternal and child health (MCH) over the past few decades [13]. In China, pregnant women are registered at the primary hospitals, and in the 32nd gestational week, they are referred to a secondary hospital or a tertiary hospital for management till delivery. And, children undergo health examinations as newborns, postnatal period, infancy, and at preschool [14]. These efforts established a strong foundation for the development of MCH in the twenty-first century, which covers both urban and rural areas [13]. In this systematic review and meta-analyses, our aim was to address the evidence gap to establish the strength of the association between maternal BMI and neonatal adverse outcomes in Chinese women.

## Methods

This systematic review and meta-analysis was performed in accordance with the recommendations of the Preferred Reporting Items for Systematic Review and Meta-analysis Protocols (PRISMA).

### Data sources and search strategy

We conducted a systematic literature search for studies on pre-pregnancy or early pregnancy BMI and the risk of perinatal health outcomes. We searched six bibliographic electronic databases (English databases: PubMed, Embase, and the Cochrane library; Chinese databases: the Chinese Journal Full Text Database of the China National Knowledge Infrastructure (CNKI), China Biology Medical Literature Database (CBM), and Wanfang DATA) to October 24, 2017. We developed search strategies comprising a combination of free-text words, words in medical subject headings and titles/abstracts for participants, exposure and study design, irrespective of publication date and follow-up duration. The integrated search strategies and results from the PubMed and CNKI databases are provided in Additional file 1. Additionally, the references of relevant reviews and included articles were searched for eligible studies. Electronic messages were sent to corresponding authors to collect extra information if the original publication lacked adequate details. We did not attempt to glean unpublished data.

### Study selection

All identified citations were exported to EndNote X7 (Thomson Reuters, USA) for management, and duplicate publications were deleted. Initially, the outcomes of the electronic searches were screened by one investigator to select potentially relevant publications according to titles and abstracts. When the title and abstract were not sufficient to determine whether the study met the uniform eligibility criteria, full texts were acquired and assessed. In the second stage, to avoid literature screening bias, two investigators independently screened the citations of the publication from the first screening to select eligible studies based on full texts. Discrepancies were resolved by consulting to a senior reviewer if necessary. Studies that were large prospective or retrospective cohorts (sample size > 1000) of pregnant Chinese women and reported BMI measures (underweight, normal weight, overweight and obesity) were included. If articles assessed the same participants, we only selected the study with a greater number of participants, the best methodological qualities and that report the most information to avoid attributing more weight to these studies in the meta-analysis. The studies defined maternal BMI categories according to multiple standards. We applied the three different standards, which were used across all regions of China, including the World Health Organization (WHO; underweight: < 18.5 kg/m^2^, normal weight: 18.5–24.9 kg/m^2^, overweight: 25–29.9 kg/m^2^ and obese: > 30 kg/m^2^), the Asia-Pacific standard (APS; underweight: < 18.5 kg/m^2^, normal weight: 18.5–22.9 kg/m^2^, overweight: 23.0–24.9 kg/m^2^ and obese: > 25.0 kg/m^2^) and the standard for Chinese adults proposed by the Working Group on Obesity in China (WGOC; underweight: < 18.5 kg/m^2^, normal weight: 18.5–23.9 kg/m^2^, overweight: 24.0–27.9 kg/m^2^ and obese: > 28.0 kg/m^2^). Birth weight was extracted from medical records or acquired by questionnaire or interview. Neonatal birth outcomes were PTB (defined as a born before 37 weeks of pregnancy, irrespective of spontaneous or medically indicated), LBW (defined as birth weight < 2500 g), SGA (defined as birth weight below the 10th percentile for gestational age), macrosomia (defined as birth weight > 4000 g), LGA (defined as birth weight above the 90th percentile for gestational age), fetal distress (defined as signs indicative of fetal hypoxia, which included fetal bradycardia, severe variable decelerations and persistent late decelerations), and neonatal asphyxia (defined as Apgar score < 7 at 1 min). Prospective or retrospective cohort studies that reported both the exposure variable (maternal BMI) and at least one of the above outcome variables were included.

### Quality appraisal

All the included articles were evaluated for quality using the Newcastle-Ottawa Quality Assessment Scale for cohort studies [15]. The scale examinest three points: the representativeness of the exposure and control groups, the comparability among groups, and follow up rates. All data were extracted independently by both investigators, and quality grades were assigned; conflicts were resolved by consensus. Studies that received scores > 7 were considered high quality; scores of 4–6 points indicated moderate quality, and scores of 0–3 points indicated low quality.

### Data extraction and management

All selected studies were read and independently abstracted by two investigators using a uniform piloted form. The following information was extracted from each study: author identification data, publication year, region of the study, sample size, study period, type of study design, source of study subjects, cut-off values for BMI categories based on standards, study outcomes and maximal adjusted potentially confounding factors. Original data for the exposed, unexposed, outcome and non-outcome categories were acquired if possible. Moreover, we extracted any reported odds ratios (*OR*s), relative risks (*RR*s) and 95% confidence intervals (95% CIs) for the hazard of maternal BMI and neonatal adverse outcomes using both unadjusted and adjusted values. Nonetheless, because of meagre data that did not provide sufficient dichotomous data on maternal BMI and neonatal birth outcomes, we limited these studies to the systematic review. An independent reviewer completed all raw data entry.

### Statistical analysis

Odds ratios and the 95% confidence interval were pooled for the dichotomous outcomes of each study. Using normal weight women as the reference group, ORs for underweight and overweight/obese women were pooled separately and forest plots were constructed. Heterogeneity among the studies was explored using the *I*^*2*^ statistic with *P* values. *I*^*2*^ values of 25, 50 and 75% were considered indicative of low, moderate and high heterogeneity, respectively, and if *I*^*2*^ > 90% pooled estimates were not calculated. Fixed-effects models were used if *I*^*2*^ < 50%; otherwise, random effects models were used to measure outcomes with heterogeneity. Publication bias was appraised through a visual inspection of funnel plots of the log OR and Egger weighted regression statistics [16]. We used the Duval and Tweedie nonparametric “trim-and-fill” procedure to further estimate the potential impact of publication bias in our meta-analysis [17].

We also performed sensitivity analyses to assess the robustness of the results (excluding each study in turn from the pooled results). All statistical analyses were carried out using Stata/SE, ver12.0 (Stata, USA). *P* values < 0.01 were considered statistically significant.

### Subgroup analysis

We conducted subgroup analysis to appraise potential sources of heterogeneity. Studies were stratified by sample size (≤10,000, 10,000–50,000 or ≥ 50,000), BMI cut-off (WHO, APS or WGOC), pre-pregnancy BMI source (recorded, measured, self-reported, questionnaire or not reported), language (English or Chinese), confounding factors (adjusted or unadjusted) and geographic region (North China, South China or All). The statistical significance of the effect modification across strata and their *P* values were calculated using random-effects meta-regression analysis.

## Results

### Description of studies

**Fig. 1:**
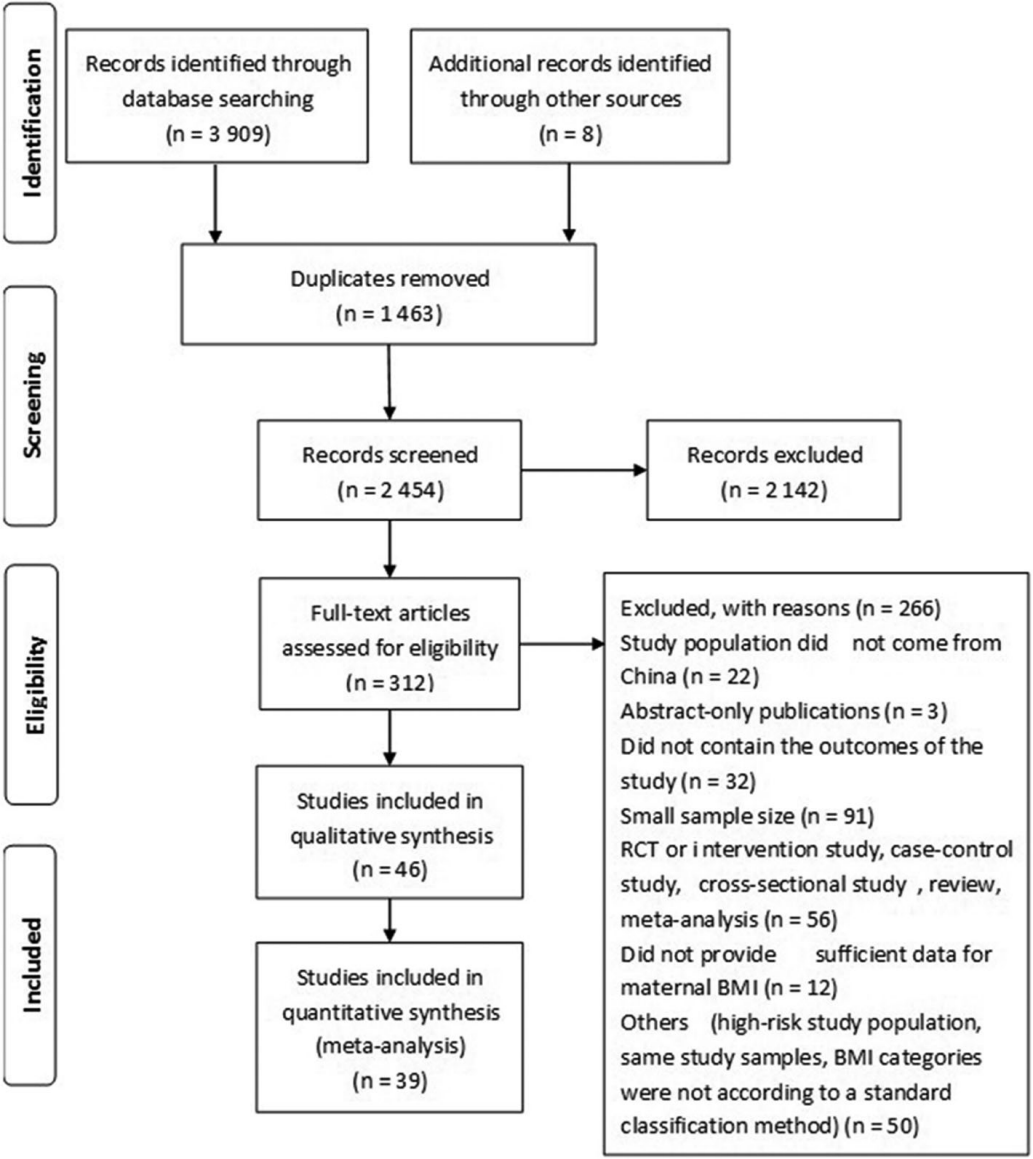
Flow diagram of the systematic selection of articles for the meta-analysis (based on PRISMA reporting)

We initially identified 3909 studies (838 in PubMed, 174 in Embase, 34 in the Cochrane Library, 1124 in CNKI, 671 in CBM, 1068 in Wanfang Database) and 8 studies from the reference list of relevant reviews. Duplicates were excluded, and 2454 unique citations underwent title and abstract screening; 312 abstracts remained for full-text article review and were further assessed for eligibility. Ultimately, forty-six unique studies were incorporated into the meta-analysis and systematic review; of these, seven studies did not provide sufficient dichotomous data and were included only in the systematically review, based on the exclusion factors listed in Fig. 1. The characteristics of the 46 included studies are presented in Additional file 2: Tables S1-S2.

The 39 studies included in the meta-analysis were published from 2005 to 2017. Regarding the category of pre-pregnancy BMI, 6 studies were based on the WHO standard [18–23], 3 were based on APS recommendations [24–26], and 30 studies were based on the WGOC standard [6, 27–55]. In terms of birth weight categories, LBW was investigated in 15 studies [6, 21, 24–27, 33, 37, 39–41, 46, 49, 52, 55], SGA in 10 studies [6, 18, 20, 28, 32, 41, 43, 45, 50, 52], macrosomia in 27 studies [6, 18, 21–26, 33, 34, 36–42, 44, 46–51, 53–55], and LGA in 7 studies [6, 20, 28, 41, 43, 45, 53]. Twenty-one studies reported preterm birth [6, 18–20, 23, 26, 28–31, 34, 35, 38, 42, 44, 45, 47–49, 52, 54], five reported fetal distress [22, 28, 31, 51, 54], and nine reported neonatal asphyxia [22, 26, 40, 42, 48–51, 54]. Twenty-three studies were of high methodological quality and received scores of 7 and above [6, 18–20, 24, 27–41, 47, 48, 52], and sixteen studies were judged to be of moderate quality [21–23, 25, 26, 42–46, 49–51, 53–55] (see Additional file 2: Table S3).

### Infant birth weight

We assessed the outcomes assessing of the 39 studies included in our meta-analysis and present them in Table1. The risk of publication bias for all outcomes is presented in Additional file 3: Figure S8-S14.

Thirty-four articles investigated the link between maternal BMI and infant birth weight. Fifteen studies evaluated the connection between maternal BMI and LBW [6, 21, 24–27, 33, 37, 39–41, 46, 49, 52, 55]. A total of 313,569 (range: 1048–85,765) subjects were included in this meta-analysis. Using mothers with normal BMI as the reference category, we found that pre-pregnancy underweight increased the risk of LBW (*OR* = 1.61, 95% CI: 1.33–1.93, *P* < 0.001; *I*^*2*^ = 86.1%). No relationship was found between LBW infants and overweight/obese mothers (*OR* = 1.18, 95% CI: 1.02–1.35, *P* = 0.022; *I*^*2*^ = 63.5%) (See Additional file 3: Figure S1).

Ten studies investigated the relationship between maternal BMI and SGA [6, 18, 20, 28, 32, 41, 43, 45, 50, 52], including a total of 172,979 (range: 1120–76,695) subjects. Our study found that compared with normal weight, maternal underweight increased the risk of SGA (*OR* = 1.75; 95% CI: 1.51–2.02; *P* < 0.001; *I*^*2*^ = 80.0%). In addition, no association was found between SGA infants and overweight/obese mothers (*OR* = 0.89; 95% CI, 0.75–1.05; *P* = 0.162; *I*^*2*^ = 78.0%) (See Additional file 3: Figure S2).

Twenty-seven studies reported the correlation between maternal BMI and macrosomia [6, 18, 21–26, 33, 34, 36–42, 44, 46–51, 53–55], totaling 303,267 (range: 1044–85,765) subjects. We pooled the data from these studies and discovered pre-pregnancy underweight was associated with a lower risk of macrosomia (*OR* = 0.49; 95% CI: 0.43–0.56; *P* < 0.001; *I*^*2*^ = 71.6%). In contrast, we found a promising association between pre-pregnancy overweight/obese and macrosomia risk compared with subjects with normal BMI (*OR* = 1.91; 95% CI, 1.75–2.09; *P* < 0.001; *I*^*2*^ = 78.7%) (See Additional file 3: Figure S3).

Seven studies evaluated the connection between maternal BMI and LGA [6, 20, 28, 41, 43, 45, 53], totaling 87,581 (range: 1419–33,973) subjects. Compared with the reference category, pre-pregnancy underweight reduced the risk of LGA (*OR* = 0.48; 95% CI: 0.39–0.59; *P* < 0.001; *I*^*2*^ = 79.7%). In contrast, the pooled *OR* of LGA was 1.88 (95% CI: 1.64–2.15; *P* < 0.001; *I*^*2*^ = 79.1%) for pre-pregnancy overweight/obese persons (see Additional file 3: Figure S4).

### Preterm birth

Twenty-one studies included were focused on maternal BMI and PTB [6, 18–20, 23, 26, 28–31, 34, 35, 38, 42, 44, 45, 47–49, 52, 54], containing 30,016 premature infants among 678,104 (range: 1044–353,477) participants. Compared with mothers with normal BMI, there was no significant association between maternal underweight and PTB in the meta-analysis (*OR* = 1.03; 95% CI, 0.95–1.15; *P* = 0.424; *I*^*2*^ = 59.1%). In contrast, significantly higher odds of PTB were found for overweight and obese combined women, and the pooled *OR* was 1.38 (95% CI, 1.25–1.52; *P* < 0.001; *I*^*2*^ = 74.8%) (see Additional file 3: Figure S5). However, the funnel plot revealed some asymmetry suggesting the probability of publication bias, the Egger test (*P* = 0.003). However, the trim and fill sensitivity analysis did not change the result, although the risk strength of the relationship was mildly attenuated (*OR* = 1.15; 95% CI, 1.04–1.27; *P* = 0.006), indicating that the correlation is not an artifact of unpublished negative studies.

### Neonatal respiratory diseases

Five cohort studies were included for assessing the relationship between maternal BMI and fetal distress [22, 28, 31, 51, 54] and 1128 fetal distress among 19,331 (range: 1160–10,251) participants. Our results revealed that maternal BMI was not significantly associated with fetal distress, whether underweight or overweight/obese mothers (See Additional file 3: Figure S6).

Nine studies included maternal BMI and neonatal asphyxia [22, 26, 40, 42, 48–51, 54] and included 465 neonatal asphyxia among 13,101 (range: 1044–3200) participants. Data from these studies were evaluated using the fixed effects model. Obese and overweight women had significantly higher odds of elevated neonatal asphyxia than normal-weight women. The pooled *OR* was 1.74 (95% CI, 1.39–2.17; *P* < 0.001; *I*^*2*^ = 0.0%). Egger test proved no evidence of publication bias (*P* = 0.483). In contrast, no significant association was revealed between low maternal BMI and neonatal asphyxia (*OR* = 1.18; 95% CI, 0.91–1.54; *P* = 0.210; *I*^*2*^ = 17.0%) (See Additional file 3: Figure S7).

### Subgroup analyses and sensitivity analyses

Moderate to high heterogeneity existed in certain perinatal and neonatal health outcomes (Table 1). Hence, we performed stratified analyses to examine the potential sources of heterogeneity for each outcome. Analyses were stratified by sample size, BMI cut-offs, BMI source, language, confounding factors and geographic region, and little evidence was found of heterogeneity between subgroups (Tables 2 and 3). The results stratified by geographic region detected an increased risk of PTB among overweight/obese mothers in North China (*OR*, 1.63; 95% CI, 1.41–1.88; *P* = 0.002) compared with South China and the country as a whole (Table 3). There was a lower risk of PTB among overweight/obese mothers in the studies with sample sizes ≥50,000 (*OR*, 1.10; 95% CI, 1.05–1.15; *P* < 0.001) compared with studies with smaller sample sizes. In terms of LGA among underweight mothers, the association was stronger in studies published in English (*OR*, 0.39; 95% CI, 0.32–0.49; *P* = 0.009) than in studies published in Chinese. Sensitivity analyses that excluded one study at a time from each pooled analysis did not show any material influence on the robustness of the results (data not shown).

**Table 1:**
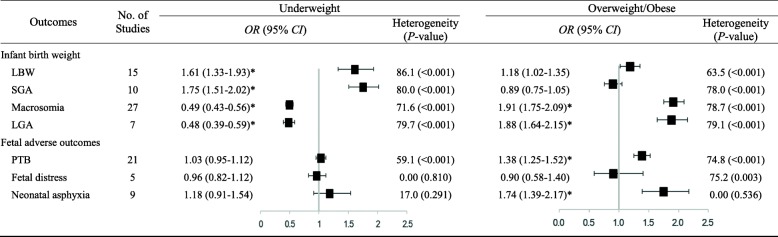
Meta-analysis summary results

* indicates statistical significance (*P* < 0.01)

**Table 2:** Analysis of infant birth weight stratified by BMI

Characteristics	LBW			SGA			Macrosomia			LGA		
	No. of Studies	Pooled *OR* (95% CI)	*P*-value	No. of Studies	Pooled *OR* (95% CI)	*P*-value	No. of Studies	Pooled *OR* (95% CI)	*P*-value	No. of Studies	Pooled *OR* (95% CI)	*P*-value
Underweight												
Sample size												
≤ 10,000	8	2.22 (1.10–4.52)	0.187	6	2.03 (1.37–3.00)	0.394	20	0.46 (0.38–0.55)	0.350	4	0.55 (0.46–0.67)	0.367
10,000~50,000	5	1.37 (1.13–1.65)		3	1.78 (1.61–1.97)		6	0.52 (0.41–0.66)		3	0.44 (0.31–0.62)	
≥ 50,000	2	1.28 (1.14–1.43)		1	1.46 (1.37–1.55)		1	0.56 (0.51–0.61)		0	NA	
BMI cut-off												
WHO	1	3.52 (1.66–7.49)	0.233	2	1.85 (0.64–2.07)	0.841	4	0.41 (0.25–0.67)	0.843	1	0.32 (0.25–0.41)	0.073
APS	3	2.14 (0.59–7.72)		0	NA		3	0.60 (0.34–1.07)		0	NA	
WGOC	11	1.43 (1.24–1.64)		8	1.74 (1.46–2.06)		20	0.49 (0.44–0.55)		6	0.52 (0.44–0.62)	
BMI source												
Recorded	11	1.72 (1.39–2.14)	0.239	4	1.88 (1.47–2.41)	0.698	18	0.51 (0.43–0.60)	0.332	3	0.50 (0.37–0.67)	0.225
Measured	1	1.30 (0.97–1.73)		2	1.53 (1.28–1.83)		1	0.54 (0.45–0.63)		2	0.59 (0.53–0.66)	
Self-reported	0	NA		1	2.28 (0.75–6.92)		3	0.48 (0.39–0.58)		0	NA	
Questionnaire	1	1.64 (0.96–2.80)		3	1.73 (1.21–2.48)		2	0.52 (0.30–0.88)		2	0.38 (0.26–0.55)	
Not reported	2	0.88 (0.34–2.23)		0	NA		3	0.37 (0.23–0.60)		0	NA	
Language												
English	3	1.31 (1.18–1.44)	0.486	5	1.70 (1.46–1.99)	0.717	5	0.50 (0.44–0.57)	0.975	3	0.39 (0.32–0.49)	0.009
Chinese	12	1.81 (1.31–2.50)		5	1.75 (1.52–2.02)		22	0.48 (0.41–0.58)		4	0.59 (0.53–0.65)	
Confounders												
Adjusted	5	1.24 (1.13–1.37)	0.307	8	1.63 (1.44–1.85)	0.022	7	0.56 (0.47–0.68)	0.127	6	0.48 (0.38–0.61)	0.967
Unadjusted	10	1.95 (1.38–2.75)		2	3.66 (1.46–9.16)		20	0.45 (0.38–0.53)		1	0.48 (0.32–0.72)	
Geographic region												
North China	6	1.31 (0.94–1.83)	0.510	4	1.82 (1.67–2.00)	0.914	12	0.45 (0.41–0.49)	0.212	3	0.43 (0.37–0.50)	0.764
South China	7	2.08 (1.41–3.05)		6	1.77 (1.43–2.20)		14	0.50 (0.40–0.63)		4	0.49 (0.36–0.68)	
All	2	1.52 (1.19–1.94)		0	NA		1	0.59 (0.51–0.67)		0	NA	
Overweight/Obesity												
Sample size												
≤ 10,000	8	1.24 (0.90–1.71)	0.718	6	1.21 (0.84–1.73)	0.135	20	2.05 (1.75–2.40)	0.284	4	1.67 (1.40–1.98)	0.162
10,000~50,000	5	1.04 (0.81–1.34)		3	0.69 (0.64–0.75)		6	1.75 (1.52–2.01)		3	2.06 (1.76–2.41)	
≥ 50,000	2	1.32 (1.04–1.68)		1	0.84 (0.75–0.94)		1	1.83 (1.68–1.99)		0	NA	
BMI cut-off												
WHO	1	1.34 (0.54–3.33)	0.731	2	1.29 (0.31–5.38)	0.733	4	1.90 (1.19–3.04)	0.776	1	2.41 (2.19–2.64)	0.067
APS	3	1.19 (0.79–1.80)		0	NA		3	1.90 (1.13–3.18)		0	NA	
WGOC	10	1.15 (0.97–1.36)		8	0.89 (0.74–1.05)		20	1.92 (1.76–2.10)		6	1.79 (1.58–2.02)	
BMI source												
Recorded	11	1.19 (1.05–1.35)	0.621	4	0.77 (0.69–0.86)	0.392	18	2.03 (1.82–2.27)	0.446	3	1.66 (1.28–2.16)	0.097
Measured	1	0.35 (0.14–0.85)		2	0.99 (0.54–1.82)		1	1.89 (1.57–2.30)		2	1.78 (1.56–2.03)	
Self-reported	0	NA		1	2.82 (1.29–6.17)		3	1.28 (0.95–1.71)		0	NA	
Questionnaire	1	2.08 (1.31–3.32)		3	0.97 (0.54–1.73)		2	1.90 (1.30–2.78)		2	2.38 (2.18–2.60)	
Not reported	2	0.92 (0.44–1.93)		0	NA		3	2.13 (1.72–2.65)		0	NA	
Language												
English	3	1.28 (1.09–1.50)	0.367	5	0.77 (0.65–0.91)	0.167	5	1.57 (1.33–1.85)	0.028	3	2.02 (1.69–2.42)	0.329
Chinese	12	1.06 (0.83–1.36)		5	1.12 (0.85–1.47)		22	2.06 (1.84–2.31)		4	1.74 (1.51–2.01)	
Confounders												
Adjusted	5	1.34 (1.08–1.65)	0.064	8	0.88 (0.73–1.05)	0.824	7	1.60 (1.37–1.88)	0.026	6	1.86 (1.61–2.15)	0.714
Unadjusted	10	1.09 (1.00–1.20)		2	1.02 (0.60–1.76)		20	2.08 (1.86–2.33)		1	2.05 (1.45–2.89)	
Geographic region												
North China	6	1.16 (0.99–1.35)	0.805	4	1.04 (0.68–1.57)	0.584	12	1.69 (1.49–1.92)	0.041	3	1.85 (1.58–2.16)	0.807
South China	7	1.28 (0.98–1.66)		6	0.84 (0.67–1.04)		14	2.18 (1.87–2.55)		4	1.88 (1.45–2.42)	
All	2	1.04 (0.80–1.35)		0	NA		1	2.18 (2.02–2.36)		0	NA	

*NA* not available, *BMI* body mass index, *WHO* World Health Organization, *APS* Asia-Pacific standard, *WGOC* Working Group on Obesity in China, *LGA* large for gestational age, *SGA* small for gestational age, *LBW* low birth weight, *PTB* premature birth

**Table 3:** Analysis of selected pregnancy outcomes stratified by BMI

Characteristics	PTB			Fetal distress			Neonatal asphyxia		
	No. of Studies	Pooled *OR* (95% CI)	*P*-value	No. of Studies	Pooled *OR* (95% CI)	*P*-value	No. of Studies	Pooled *OR* (95% CI)	*P*-value
Underweight									
Sample size									
≤ 10,000	13	0.92 (0.72–1.16)	0.344	4	1.05 (0.80–1.13)	0.505	9	1.24 (0.92–1.67)	–
10,000~50,000	6	1.10 (0.99–1.22)		1	0.93 (0.77–1.12)		0	NA	
≥ 50,000	2	1.06 (1.00–1.12)		0	NA		0	NA	
BMI cut-off									
WHO	4	1.17 (0.99–1.38)	0.240	1	1.40 (0.60–3.30)	0.445	1	1.57 (0.62–3.97)	0.956
APS	1	1.05 (0.68–1.62)		0	NA		1	0.90 (0.41–1.97)	
WGOC	16	0.99 (0.91–1.09)		4	0.95 (0.81–1.12)		7	1.26 (0.87–1.84)	
BMI source									
Recorded	10	1.00 (0.82–1.20)	0.775	3	1.03 (0.78–1.38)	0.674	7	1.30 (0.90–1.88)	0.569
Measured	4	1.05 (0.91–1.22)		0	NA		0	NA	
Self-reported	4	0.92 (0.68–1.24)		1	0.93 (0.77–1.12)		0	NA	
Questionnaire	2	1.20 (1.03–1.39)		1	1.52 (0.43–5.31)		1	1.81 (0.60–5.48)	
Not reported	1	0.94 (0.69–1.27)		0	NA		1	0.91 (0.54–1.54)	
Language									
English	10	1.03 (0.95–1.12)	0.688	2	0.93 (0.78–1.10)	0.388	0	NA	–
Chinese	11	1.04 (0.84–1.29)		3	1.14 (0.79–1.64)		9	1.24 (0.92–1.67)	
Confounders									
Adjusted	12	1.01 (0.93–1.10)	0.276	2	0.93 (0.78–1.10)	0.388	0	NA	–
Unadjusted	9	1.12 (0.89–1.40)		3	1.14 (0.79–1.64)		9	1.24 (0.92–1.67)	
Geographic region									
North China	8	0.79 (0.62–1.01)	0.083	2	0.99 (0.65–1.50)	0.901	2	1.14 (0.59–2.20)	0.875
South China	10	1.13 (0.98–1.30)		3	0.96 (0.81–1.14)		7	1.25 (0.87–1.81)	
All	3	1.06 (1.01–1.12)		0	NA		0	NA	
Overweight/Obesity									
Sample size									
≤ 10,000	13	1.64 (1.46–1.83)	< 0.001	4	0.94 (0.50–1.77)	0.869	9	1.74 (1.38–2.18)	–
10,000~50,000	6	1.28 (1.13–1.45)		1	0.85 (0.60–1.19)		0	NA	
≥ 50,000	2	1.10 (1.05–1.15)		0	NA		0	NA	
BMI cut-off									
WHO	4	1.40 (1.14–1.79)	0.775	1	0.92 (0.44–1.92)	0.997	1	1.08 (0.49–2.39)	0.227
APS	1	1.39 (0.78–2.49)		0	NA		1	1.42 (0.54–3.71)	
WGOC	16	1.37 (1.23–1.54)		4	0.91 (0.54–1.52)		7	1.84 (1.44–2.34)	
BMI source									
Recorded	10	1.29 (1.14–1.47)	0.245	3	0.65 (0.49–0.86)	0.097	7	1.72 (1.32–2.24)	0.830
Measured	4	1.24 (0.99–1.56)		0	NA		0	NA	
Self-reported	4	1.79 (1.44–2.24)		1	0.85 (0.60–1.19)		0	NA	
Questionnaire	2	1.42 (0.93–2.19)		1	2.63 (1.37–5.03)		1	2.76 (1.50–5.08)	
Not reported	1	1.42 (1.01–1.99)		0	NA		1	1.11 (0.60–2.07)	
Language									
English	10	1.39 (1.23–1.56)	0.839	2	0.74 (0.56–0.98)	0.533	0	NA	–
Chinese	11	1.37 (1.18–1.60)		3	1.10 (0.44–2.79)		9	1.74 (1.38–2.18)	
Confounders									
Adjusted	12	1.38 (1.23–1.55)	0.983	2	0.74 (0.56–0.98)	0.533	0	NA	–
Unadjusted	9	1.46 (1.29–1.52)		3	1.10 (0.44–2.79)		9	1.74 (1.38–2.18)	
Geographic region									
North China	8	1.63 (1.41–1.88)	0.002	2	1.26 (0.31–5.07)	0.472	2	2.21 (1.19–4.09)	0.283
South China	10	1.26 (1.12–1.41)		3	0.77 (0.59–1.01)		7	1.63 (1.26–2.09)	
All	3	1.11 (1.06–1.16)		0	NA		0	NA	

*NA* not available, *BMI* body mass index, *WHO* World Health Organization, *APS* Asia-Pacific standard, *WGOC* Working Group on Obesity in China, *LGA* large for gestational age, *SGA* small for gestational age, *LBW* low birth weight, *PTB* premature birth

### Narrative summary of papers not included in the meta-analysis

The remaining 7 studies were excluded from the meta analyses due to a lack of sufficient dichotomous data [56–62]. Table 4 presented results of the seven studies, most of which were consistent with our analysis. However, a dramatic association between pre-pregnancy underweight and risk of PTB (*AOR* = 1.16; 95% CI, 1.08–1.25) was reported in a prospective cohort study of 536,098 women who were enrolled in the National Free Preconception Health Examination Project (NFPHEP) in China [58], which was contrary to our results.

**Table 4:** Narrative summary of papers not included in the meta-analysis

Study	Sample	Study design	BMI categories	Outcomes	Odd ratios (95% CI)		
					Underweight	Overweight	Obese
Lei et al. [56]	5535	Prospective cohort study	APS	PTB	1.00(0.77, 1.30)	1.54(1.14, 2.08)	1.42(1.02, 1.97)
				SGA	1.62(1.13, 2.34)	0.71(0.38, 1.34)	0.69(0.34, 1.37)
				LGA	0.65(0.52, 0.82)	1.32(1.02, 1.71)	2.35(1.81, 3.04)
				Neonatal asphyxia	1.60(0.80, 3.19)	0.99(0.34, 2.85)	2.38(1.06, 5.30)
Du et al. [57]	3772	Retrospective cohort study	WGOC	Macrosomia	0.38(0.21, 0.68)	2.90(1.99, 4.23)	6.3(3.42, 11.47)
				SGA	1.86(1.39, 2.50)	0.43(0.25, 0.74)	0.54(0.21, 1.40)
				LGA	0.41(0.27, 0.63)	2.23(1.66, 2.99)	3.99(2.41, 6.60)
Pan et al. [58]	536,098	Prospective cohort study	WGOC	PTB	1.16 (1.08, 1.25)	1.01(0.92, 1.10)	1.18 (0.99, 1.4)
				LBW	1.57 (1.4, 1.77)	1.22 (1.05, 1.42)	1.60 (1.2, 2.12)
Li et al. [59]	48,867	Retrospective cohort study	WHO	SGA	1.66 (1.44, 1.91)	0.73 (0.59, 0.91)	1.09 (0.67, 1.77)
				LGA	0.54 (0.47, 0.62)	2.55 (2.32, 2.80)	3.95 (3.20, 4.87)
Bao et al. [60]	13,711	Retrospective cohort study	WHO	Macrosomia	–	2.17 (1.60, 2.94)	5.81 (4.26, 7.92)
Huang et al. [61]	3081	Prospective cohort study	WGOC	SGA	1.67 (1.25, 2.23)	0.91 (0.56, 1.47)	
				LGA	0.61 (0.44, 0.83)	1.50 (1.11, 2.04)	
Jiang et al. [62]	2241	Retrospective cohort study	WGOC	PTB	–	1.18 (0.41, 2.12)	2.35 (0.89, 3.98)
				SGA	–	0.76 (0.45, 0.99)	1.02 (0.67, 1.76)
				LGA	–	1.56 (1.01, 2.31)	1.71 (1.07, 3.20)
				Fetal distress	–	0.67 (0.34, 1.39)	0.31 (0.08, 1.35)

*BMI* body mass index, *WHO* World Health Organization, *APS* Asia-Pacific standard, *WGOC* Working Group on Obesity in China, *LGA* large for gestational age, *SGA* small for gestational age, *LBW* low birth weight, *PTB* premature birth

## Discussion

This systematic review and meta-analysis of over 1.6 million Chinese mothers examined the quantitative effect of maternal BMI on adverse neonatal outcomes. In summary, maternal overweight or obesity is associated with macrosomia, LGA, PTB and neonatal asphyxia, while maternal underweight is associated with LBW and SGA.

The analysis results showed that there was a positive correlation between maternal overweight/obesity and excessive fetal growth. Mothers who are overweight or obese during gestation have infants that are more likely to have macrosomia or be LGA, which increases the risk of several delivery complications, such as shoulder dystocia, surgical deliveries and severe perineal tears [63]. In addition, underweight women have an increased risk of delivering infants with SGA and LBW. Therefore, both extremes of maternal BMI range may influence the risk of neonatal adverse outcomes.

PTB is the leading cause of neonatal morbidity and mortality [63]. Rahman et al. [64] and Liu et al. [65] quantified the association between maternal BMI and perinatal outcomes in low and middle income countries and found that overweight and obesity were not associated with preterm delivery and that women who were underweight had a higher risk of preterm delivery. However, in our study and several observational studies, neonates born to women who are overweight or obese were more likely to be born preterm, a condition that was not associated with underweight mothers. Additionally, women who are overweight or obese have an increased risk of premature rupture of membrane (PROM) and other complications, which leads to higher rates of iatrogenic PTB [66]. Because potential disparities could not be ruled out completely, these results need to be verified by further studies.

Neonatal asphyxia is defined as the state producing a combination of systemic hypoxemia, hypercapnia, and metabolic acidosis that may occur before and during birth, and during neonatal period [67]. The present study is the first attempt to assess the pooled risk of neonatal respiratory diseases attributable to maternal BMI. Our research indicated that the incidence of neonatal asphyxia in infants whose mothers were overweight or obese was generally higher than that of the comparison group. This is consistent with the results of a population based study [68]. However, asphyxia in newborn infants causes cerebrovascular disease and neonatal brain injury and may lead to lifelong neurodevelopmental disabilities [69].

### Strengths and limitations

We examined prospective and retrospective cohort studies with large sample sizes, including many well-designed high-quality studies, that included Chinese sources. Furthermore, we followed the checklist of the PRISMA, which includes a comprehensive search strategy, assessment of publication bias, and heterogeneity testing with a stratified analysis to explore the impact of maternal BMI measured or obtained before or during the first trimester on birth outcomes.

However, several limitations need to be mentioned. One potential limitation is that only published studies were searched. As we know, studies with optimistic results may be published more easily than those with unfavorable results. Although there was publication bias regarding PTB among overweight/obese mothers, sensitivity analyses using the trim-and-fill method showed that the result was reliable. Nevertheless, this method does not entirely exclude the possibility of publication bias. Second, moderate to high heterogeneity was observed for some adverse outcomes. On the one hand, the studies included in this meta-analysis used varied BMI categories, which might have resulted in heterogeneity. On the other hand, we obtained narrow positive or negative ORs for the comparisons because of the large number of papers and subjects included in our study. Third, the impact of potential confounding on the results was not considered during the study.

## Conclusions

An analysis of the current evidence in the literature suggests that maternal weight status is critically important to neonatal health during the perinatal period. In summary, maternal overweight/obesity is associated with macrosomia, LGA, PTB and neonatal asphyxia, while maternal underweight is associated with LBW and SGA. Management of weight during pregnancy might help reduce their adverse neonatal outcomes in future intervention studies or programmes. In the meantime, we recommend intervention developers and behavior change agents in the field to developing tailored interventions within women of childbearing age.

## Additional files

Additional file 1: Search strategies and results for the PubMed and CNKI. (DOC 96 kb)

Additional file 2: Data extraction and quality assessment. (DOC 397 kb)

Additional file 3: Forest plot and funnel plot of the association between maternal BMI and neonatal adverse outcomes. (DOC 167 kb)

## Abbreviations

APS: Asia-Pacific standard
BMI: Body mass index
LBW: Low birth weight
LGA: Large for gestational age
PROM: Premature rupture of membrane
PTB: Premature birth
SGA: Small for gestational age
WGOC: Working Group on Obesity in China
