# Supplementary material for: Maternal body mass index and risk of neonatal adverse outcomes in China: a systematic review and meta-analysis
Source: BMC Pregnancy Childbirth. 2019 Mar 29;19:105. doi: 10.1186/s12884-019-2249-z (PMC6440121; doi:10.1186/s12884-019-2249-z)
Supplement: Supplementary file 1 — Search strategies and results for the PubMed and CNKI. (DOC 96 kb) [file 12884_2019_2249_MOESM1_ESM.doc]

**Additional file 1** Search strategies and results for the PubMed (accessed on 2017/10/24)

|  | **Query** | **Result** |
| --- | --- | --- |
| #1 | Body Mass Index [MeSH Terms] | 99477 |
| #2 | body mass index [Title/Abstract] | 136822 |
| #3 | Body Weight [MeSH Terms] | 395277 |
| #4 | body weight [Title/Abstract] | 175180 |
| #5 | weight gain [Title/Abstract] | 50246 |
| #6 | Overweight [MeSH Terms] | 173369 |
| #7 | overweight [Title/Abstract] | 51806 |
| #8 | Obesity [MeSH Terms] | 169094 |
| #9 | obesity [Title/Abstract] | 186760 |
| #10 | obese [Title/Abstract] | 100283 |
| #11 | #1 OR #2 OR #3 OR #4 OR #5 OR #6 OR #7 OR #8 OR #9 OR #10 | 704886 |
| #12 | Pregnant Women [MeSH Terms] | 5987 |
| #13 | mother* [Title/Abstract] | 181290 |
| #14 | Pregnancy [MeSH Terms] | 804449 |
| #15 | pregnan* [Title/Abstract] | 446060 |
| #16 | gravid [Title/Abstract] | 4549 |
| #17 | obstetric [Title/Abstract] | 35776 |
| #18 | antenatal [Title/Abstract] | 28347 |
| #19 | antepartum [Title/Abstract] | 5008 |
| #20 | gestation* [Title/Abstract] | 173466 |
| #21 | #12 OR #13 OR #14 OR #15 OR #16 OR #17 OR #18 OR #19 OR #20 | 1051719 |
| #22 | Cohort Studies [MeSH Terms] | 1602115 |
| #23 | Longitudinal Studies [MeSH Terms] | 103717 |
| #24 | Follow-Up Studies [MeSH Terms] | 560572 |
| #25 | Prospective studies [MeSH Terms] | 433716 |
| #26 | Case-Control Studies [MeSH Terms] | 826296 |
| #27 | Retrospective Studies [MeSH Terms] | 616761 |
| #28 | cohort* [Title/Abstract] | 397676 |
| #29 | longitudinal [Title/Abstract] | 185722 |
| #30 | prospective [Title/Abstract] | 447624 |
| #31 | retrospective [Title/Abstract] | 378173 |
| #32 | incidence stud* [Title/Abstract] | 1324 |
| #33 | follow up [Title/Abstract] | 762976 |
| #34 | case control [tw] | 261402 |
| #35 | #22 OR #23 OR #24 OR #25 OR #26 OR #27 OR #28 OR #29 OR #30 OR #31 OR #32 OR #33 OR #34 | 2598069 |
| #36 | meta-analysis [MeSH term] | 15322 |
| #37 | Review [Publication Type] | 2227285 |
| #38 | #36 OR #37 | 2237747 |
| #39 | #35 NO #38 | 2454082 |
| #41 | China [Affiliation] | 1004680 |
| #42 | China [MeSH Terms] | 131838 |
| #43 | China [Title/Abstract] | 112992 |
| #44 | "People's Republic of China" [Title/Abstract] | 2598 |
| #45 | "Mainland China" [Title/Abstract] | 2756 |
| #46 | Manchuria [Title/Abstract] | 74 |
| #47 | Sinkiang [Title/Abstract] | 21 |
| #48 | "Inner Mongolia" [Title/Abstract] | 1296 |
| #49 | #41 OR #42 OR #43 OR #44 OR #45 OR #46 OR #47 OR #48 | 1070158 |
| **#50** | **#11 AND #21 AND #35 AND #39 AND #49** | **838** |

**Additional file 2** Search strategies and results for the CNKI (accessed on 2017/10/24)

|  | **Query** | **Result** |
| --- | --- | --- |
| #1 | ‘Fei Pang’ (which means obesity) [Subject Term] | 46770 |
| #2 | ‘Fei Pang’ (which means obesity) [Keyword] | 12526 |
| #3 | ‘Chao Zhong’ (which means overweight) [Subject Term] | 10067 |
| #4 | ‘Chao Zhong’ (which means overweight) [Keyword] | 1985 |
| #5 | ‘Ti Zhong Zeng Jia’ (which means weight gain) [Subject Term] | 3587 |
| #6 | ‘Ti Zhong Zeng Jia’ (which means weight gain) [Keyword] | 927 |
| #7 | BMI [Subject Term] | 23160 |
| #8 | BMI [Keyword] | 10526 |
| #9 | #1 OR #2 OR #3 OR #4 OR #5 OR #6 OR #7 OR #8 | 69043 |
| #10 | ‘Yun Fu’ (which means pregnancy woman) [Subject Term] | 67061 |
| #11 | ‘Yun Fu’ (which means pregnancy woman) [Keyword] | 35611 |
| #12 | ‘Huai Yun’ (which means pregnancy) [Subject Term] | 10973 |
| #13 | ‘Huai Yun’ (which means pregnancy) [Keyword] | 99 |
| #14 | ‘Ren Shen’ (which means gestation) [Subject Term] | 194910 |
| #15 | ‘Ren Shen’ (which means gestation) [Keyword] | 22664 |
| #16 | ‘Ren Shen Jie Ju’ (which means pregnancy outcomes) [Subject Term] | 18884 |
| #17 | ‘Ren Shen Jie Ju’ (which means pregnancy outcomes) [Keyword] | 13025 |
| #18 | ‘Bu Liang Ren Shen Jie Ju’ (which means adverse pregnancy outcomes) [Subject Term] | 1852 |
| #19 | ‘Bu Liang Ren Shen Jie Ju’ (which means adverse pregnancy outcomes) [Keyword] | 364 |
| #20 | #12 OR #13 OR #14 OR #15 OR #16 OR #17 OR #18 OR #19 OR #20 | 240034 |
| #21 | ‘meta Fen Xi’ (which means meta-analysis) [Subject Term] | 24725 |
| #22 | ‘meta Fen Xi’ (which means meta-analysis) [Keyword] | 18156 |
| #23 | ‘Zong Shu’ (which means review) [Subject Term] | 309606 |
| #24 | ‘Zong Shu’ (which means review) [Keyword] | 60798 |
| #25 | #36 OR #37 | 379526 |
| **#27** | **#9 AND #20 NO #25** | **1124** |
