# Supplementary material for: Maternal body mass index and risk of neonatal adverse outcomes in China: a systematic review and meta-analysis
Source: BMC Pregnancy Childbirth. 2019 Mar 29;19:105. doi: 10.1186/s12884-019-2249-z (PMC6440121; doi:10.1186/s12884-019-2249-z)
Supplement: Supplementary file 2 — Data extraction and quality assessment. (DOC 397 kb) [file 12884_2019_2249_MOESM2_ESM.doc]

**Additional file 2 Data extraction and quality assessment**

**Table S1** Characteristics of studies examining the relationship between pre-pregnancy BMI and BW

| **Source** | **province**  **study period** | **Study design/source of population (n)** | **Source of BMI** | **BMI categories** | **Outcomes** | **Maximum adjustment available** |
| --- | --- | --- | --- | --- | --- | --- |
| Chen *et al*. (2013) | 14 provinces of China  2011 | Retrospective cohort from 39 hospitals database (n=101,163) | Recorded from medical records | WGOC | LBW | NR |
| Liu *et al*. (2015) | Beijing 10/2013–10/2014 | Retrospective cohort from the Beijing Friendship Hospital (n=2,973) | Self-reported | WHO | Cesarean deliveries, PPH, PTB, PPROM, GDM, GHT, Preeclampsia  SGA, Macrosomia | Maternal age, height, gestational weeks, family history of hypertension, family history of diabetes and birth weight |
| Liu *et al*. (2011) | Liaoning 2007–2009 | Retrospective cohort from three hospitals of Shenyang (n=5,047) | Recorded from medical records | WGOC | GDM, PROM, cesarean delivery, and others; PTB, fetal distress, LGA, SGA, stillbirth | Maternal age, education and gestational weight gains |
| Wang *et al*. (2015) | 18 provinces of China  1/2010–12/2010 | Retrospective cohort from the free National Pre-pregnancy Checkups Project (NPCP) (n=172,206) | Measured during the physical examination | WGOC | PTB | Maternal age, education level, ethnic group, and career, hypertension, anemia, alcohol use |
| Wang *et al*. (2011) | Hebei, Jiangsu, Zhejiang  1993–2005 | Retrospective cohort from the Perinatal Health Care Surveillance System (PHCSS) (n=353,477) | Recorded from medical records | WGOC | PTB | Maternal age, education, occupation, city or county, gender of infant, and year of delivery |
| Ding *et al*. (2015) | Anhui 11/2008–10/2010 | Prospective cohort from the China-Anhui Birth Cohort Study (C-ABCS) (n=10,251) | self-reported | WGOC | GDM, PROM, caesarean delivery, PTB, fetal distress | Maternal age, area of residence, maternal education, economic status, parity, maternal height, cigarette smoking and drinking |
| Zhang *et al*. (2016) | Hubei 6/2011-6/2013 | Retrospective cohort from the Wuhan Maternal and Child Health Management Information System (WMCHMIS) (n=76,695) | Recorded from medical records | WGOC | SGA, PTB | Maternal age, occupation, education, gravidity, parity, gender of baby, season at conception |
| Yang *et al.* (2015) | Hubei  6/2011–6/2013 | Retrospective cohort from the perinatal  health care system (n=85,765) | Recorded from medical records | WGOC | LBW, Macrosomia | Maternal age, maternal education, infant gender |
| Huang *et al.* (2016) | Hebei, Liaoning, Hunan, Fujian, Sichuan, Yunnan, 10/2013–9/2014 | Prospective cohort study from Maternal and Newborn’s Health Monitoring System (MNHMS) (n=17,475) | Measurements obtained during  the first antenatal visit | WHO | PTB | Maternal pre-pregnancy BMI, districts, type of residents, maternal ethnicity, maternal education, maternal age, parity and infant sex |
| Wei *et al*. (2015) | Beijing  6/2013–11/2013 | Prospective cohort from 15 medical centers in Beijing (n=14,451) | Self-reported | WGOC | Macrosomia, PTB, GDM, Cesarean section, Neonatal NICU, Preeclampsia | NR |
| Leung *et al*. (2008) | Hong Kong  1995–2005 | Retrospective cohort from the Obstetric Specialty Clinical Information System (OBSCIS) (n=29,303) | Questionnaire | WHO | GDM, PTB, SGA, LGA, Stillbirth, Neonatal death, caesarean section | Maternal age, parity, gestational age, caesarean section, DM |
| Hu *et al*. (2012) | Jiangsu  2001–2009 | Prospective cohort from Perinatal Monitoring System of Maternal and Child Health Care Hospital of Kunshan (n=33,039) | Recorded from medical records | APS | LBW, Macrosomia | Maternal age, educational level, gestational weeks, parity and infant sex |
| Zhang *et al*. (2016) | Hubei  2012–2014 | Retrospective cohort from Wuhan Medical and Health Center for Women and Children (n=11,020) | Measurements obtained during  the first antenatal visit | WGOC | PTB | Maternal age, GDM, Maternal age, occupation, economic status, hypertension status |
| Yang *et al.* (2012) | Beijing  2005 and 2009 | Retrospective cohort from Peking University First Hospital (n=4,736) | Recorded from medical records | WHO | LBW, Cesarean section, macrosomia | NR |
| Ren *et al*. (2016) | Beijing 6/2013–11/2013 | Retrospective cohort study from 15 hospitals (n=13,938) | Questionnaire | WGOC | Macrosomia | NR |
| Wang *et al*. (2013) | Beijing 1/2010–12/2010 | Retrospective cohort study from Beijing Obstetrics and Gynecology Hospital and Haidian Maternity and Child Health Care Hospital (n=16,460) | Recorded from medical records | WGOC | LBW, macrosomia | NR |
| Li *et al*. (2013) | Tianjin 6/2009–5/2011 | Retrospective cross-sectional study from Tianjin Maternal and Newborn’s Health Monitoring System (n= 33,973) | Recorded from health care records | WGOC | LBW, PTB, SGA, LGA, Cesarean section, GDM, PIH | Maternal age, marital status, households income, smoking, employment status and others |
| Chen *et al*. (2010) | Liaoning 1/2009–6/2009 | Retrospective cohort from 2 hospital clinics (n=2,586) | Self-reported | WGOC | PTB, GDM, Preeclampsia, PIH, macrosomia | Age, education, employment status, parity, family history of diabetes and hypertension |
| Zhang *et al*. (2015) | Beijing 1/2012–12/2013 | Retrospective cohort from 1 hospital clinic (n= 4,036) | NR | WGOC | LBW, macrosomia | NR |
| Zhu *et al*. (2010) | Zhejiang 1/2007–12/2008 | Retrospective cohort from 1 hospital clinic (n= 1,160) | Recorded from medical records | WHO | Macrosomia, GDM, dystocia, neonatal asphyxia | NR |
| Liu *et al*. (2015) | Beijing  1/2014–9/2014 | Retrospective cohort from 1 hospital clinic (n= 2,124) | Recorded from medical records | WGOC | GDM, PPROM, PTB, cesarean section, PIH, macrosomia, postpartum hemorrhage, preeclampsia | NR |
| Li *et al*. (2016) | Henan  2013–2015 | Retrospective cohort from 1 hospital clinic (n=1,456) | Recorded from medical records | APS | GDM, PPROM, PTB, LBW, PIH, macrosomia, cesarean section, neonatal asphyxia | NR |
| Yang *et al*. (2012) | Fujian 1/2009–12/2011 | Retrospective cohort from 1 hospital clinic (n= 1,044) | Recorded from medical records | WGOC | Cesarean section, GDM, PTB, macrosomia, PIH, neonatal asphyxia | NR |
| Kong *et al*. (2014) | Jiangxi 1/2011–10/2013 | Retrospective cohort from maternal and child health hospital (n= 1,048) | Recorded from medical records | WGOC | Cesarean section, PTB, LBW, macrosomia, GDM fetal distress, | NR |
| Zhu *et al*. (2013) | Zhejiang 7/2011–6/2012 | Retrospective cohort from maternal and child health hospital (n=4,749) | Recorded from medical records | WHO | PTB, Cesarean section, PIH, macrosomia | NR |
| Luo et al. (2005) | Guangxi  1/2002–12/2004 | Retrospective cohort from 1 hospital clinic (n= 1,120) | Recorded from medical records | WGOC | PIH, GDM, macrosomia, Cesarean section, SGA, neonatal asphyxia, postpartum hemorrhage | NR |
| Xu *et al*. (2014) | Zhejiang  1/2011–12/2011 | Retrospective cohort from maternal and child health hospital (n= 1,530) | Recorded from medical records | APS | LBW, cesarean section, macrosomia, BW | NR |
| Li *et al*. (2014) | 14 provinces of China 1/2011–12/2011 | Retrospective study from 39 hospital clinics (n= 48,867) | Recorded from medical records | WGOC | PIH, GDM, macrosomia, postpartum hemorrhage, LBW, PPROM, neonatal death | NR |
| Xia *et al*. (2012) | Jiangxi 8/2010–10/2011 | Retrospective cohort from 1 hospital clinic (n=1,200) | Recorded from medical records | WGOC | GDM, Cesarean section, PIH, fetal distress, macrosomia, postpartum hemorrhage, LBW | NR |
| Chu *et al*. (2012) | Anhui 10/2008–10/2010 | Prospective cohort from Eight maternal and child health care institutions  (n=12,355) | Measurements obtained during  the first antenatal visit | WGOC | SGA, LGA, LBW macrosomia | Maternal age, marital status, households income and others |
| Li et al. (2016) | Guangdong  3/2012–3/2015 | Retrospective cohort from 1 hospital clinic (n= 3,200) | NR | WGOC | PIH, GDM, macrosomia, Cesarean section, neonatal asphyxia, postpartum hemorrhage, PTB, birth defects | NR |
| Wang *et al*. (2011) | Anhui  1/2008–12/2009 | Prospective cohort from 1 hospital clinic (n= 1,419) | Questionnaire | WGOC | SGA, LGA | NR |
| Liu *et al*. (2015) | Henan  9/2012–9/2013 | Prospective cohort from 1 hospital clinic  (n=1,245) | Questionnaire | WGOC | GDM, PIH, macrosomia, cesarean section, neonatal asphyxia, fetal distress | NR |
| Sun *et al*. (2016) | Jiangsu 6/2014–2/2015 | Retrospective study from maternal and child health care institution (n= 3,943) | Questionnaire | WGOC | PTB, LBW, SGA | Maternal age, marital status, households income, smoking, employment status and others |
| Tong *et al*. (2013) | Nanjing 1/2009–4/2010 | Retrospective cohort from 1 hospital clinic (n= 2,409) | Recorded from medical records | WGOC | GDM, PIH, macrosomia, cesarean section, PTB, FGR | NR |
| Fan et al. (2015) | Hainan 4/2010–4/2014 | Retrospective cohort from 1 hospital clinic (n= 1,743) | Recorded from medical records | WGOC | Macrosomia, LGA | Maternal age and others |
| Liu *et al*. (2010) | Liaoning 2007–2009 | Retrospective cohort from 3 hospital clinics (n=3,741) | Measurements obtained during  the first antenatal visit | WGOC | GDM, Cesarean section, PTB, preeclampsia, LGA, SGA | Maternal age and others |
| Li et al. (2012) | Anhui  8/2011–7/2012 | Retrospective cohort from 1 hospital clinic (n= 1,628) | Recorded from medical records | WGOC | FGR, GDM, macrosomia, preeclampsia, neonatal asphyxia, postpartum hemorrhage, PTB, PPROM | NR |
| Xue *et al*. (2014) | Beijng  10/2012–10/2013 | Retrospective cohort from 1 hospital clinic (n=3,840) | NR | WGOC | GDM, Cesarean section, PIH, preeclampsia, LBW, macrosomia | NR |

NR, not reported; BMI, body mass index; BW, birth weight; WHO, World Health Organization; APS, Asia-Pacific standard; WGOC, Working Group on Obesity in China; LGA, large for gestational age; SGA, small for gestational age; LBW, low birth weight; PTB, premature birth.

**Table S2** Characteristics of studies in the narrow review

| **Source** | **province**  **study period** | **Study design/source of population (n)** | **Source of BMI** | **BMI categories** | **Outcomes** | **Maximum adjustment available** |
| --- | --- | --- | --- | --- | --- | --- |
| Lei *et al*. (2016) | Guangdong  1/2012–12/2014 | Prospective cohort from the Department of Obstetrics of Guangdong Women and Children Hospital (n=5,535) | Self-reported | APS | PE, GDM, PTB, SGA or LGA, Neonatal asphyxia, Fetal demise, | Maternal age, parity |
| Du *et al*. (2017) | Zhejiang 10/2013–7/2014 | Retrospective cohort from Ninghai Maternity and Child Health Care Hospital (n=3,772) | Recorded from medical records | WGOC | LBW, SGA, LGA, Macrosomia | Maternal age, gestational age, height, and gender |
| Pan *et al*. (2016) | 31 provinces of China  2010–2012 | Prospective cohort study from the National Free Preconception Health Examination Project (NFPHEP) (n=536,098) | Obtained from the pre-pregnancy examinations | WGOC | PTB, LBW, SM, SB | Maternal age, ethnicity, educational level, occupation, smoking, alcohol intake, a previous history of pregnancy |
| Li *et al*. (2015) | 14 provinces of China  1/2011–12/2011 | Retrospective cohort from multicenter (n=48,867) | Clinical medical records | WHO | PIH, GDM, postpartum hemorrhage, cesarean delivery, SGA, LGA | Maternal age, smoking, alcohol consumption, maternal height, social status, residential area and gestational weight gain |
| Bao *et al.* (2010) | Heilongjiang 1/2001–12/2005 | Retrospective population cohort from 16 hospitals (n=13,711) | Recorded from medical records | WHO | Macrosomia | NR |
| Huang *et al*. (2016) | Anhui 5/2013–9/2014 | Prospective cohort from the Ma Anshan maternity and child care hospital (n=3,081) | Measured during the physical examination | WGOC | LGA, SGA | Maternal age, educational level, gestational weeks, parity and infant sex, and others |
| Jiang *et al*. (2013) | Henan  3/2010–3/2012 | Retrospective cohort from 1 hospital clinic (n=2,241) | Recorded from medical records | WGOC | PTB, macrosomia, LGA, SGA, fetal distress | NR |

NR, not reported; BMI, body mass index; BW, birth weight; WHO, World Health Organization; APS, Asia-Pacific standard; WGOC, Working Group on Obesity in China; LGA, large for gestational age; SGA, small for gestational age; LBW, low birth weight; PTB, premature birth.

**Table S3** Quality assessment (grade) of the studies included in the meta-analysis

| **Identification** | **Final quality** | **Score** | **Selection** | | | | | **Comparability** | | **Outcome** | | |
| --- | --- | --- | --- | --- | --- | --- | --- | --- | --- | --- | --- | --- |
|  |  |  | **1** | **2** | | **3.** | **4** | **5A** | **5B** | **6** | **7** | **8** |
|  |  |  | Exposed cohort truly representative | Nonexposed cohort drawn from the same community | | Ascertainment of exposure | Outcome of interest not present at start | Cohorts comparable on basis of age | Cohorts comparable on other factor(s) | Quality of outcome assessment | Follow-up long enough for outcomes to occur | Complete accounting for cohorts |
| Chen et al. 2013 | High | 7 | 1 | | 1 | 1 | 1 | 0 | | 1 | 1 | 1 |
| Liu et al. 2015 | High | 8 | 1 | | 1 | 0 | 1 | 2 | | 1 | 1 | 1 |
| Lei et al. 2016 | High | 9 | 1 | | 1 | 0 | 1 | 2 | | 1 | 1 | 1 |
| Liu et al. 2011 | High | 9 | 1 | | 1 | 1 | 1 | 2 | | 1 | 1 | 1 |
| Du et al. 2017 | High | 9 | 1 | | 1 | 1 | 1 | 2 | | 1 | 1 | 1 |
| Wang et al. 2015 | High | 9 | 1 | | 1 | 1 | 1 | 2 | | 1 | 1 | 1 |
| Pan et al. 2016 | High | 9 | 1 | | 1 | 1 | 1 | 2 | | 1 | 1 | 1 |
| Li et al. 2015 | High | 9 | 1 | | 1 | 1 | 1 | 2 | | 1 | 1 | 1 |
| Wang et al. 2011 | High | 9 | 1 | | 1 | 1 | 1 | 2 | | 1 | 1 | 1 |
| Ding et al. 2015 | High | 9 | 1 | | 1 | 1 | 1 | 2 | | 1 | 1 | 1 |
| Zhang et al. 2016 | High | 9 | 1 | | 1 | 1 | 1 | 2 | | 1 | 1 | 1 |
| Yang et al. 2015 | High | 9 | 1 | | 1 | 1 | 1 | 2 | | 1 | 1 | 1 |
| Huang et al.2016 | High | 9 | 1 | | 1 | 1 | 1 | 2 | | 1 | 1 | 1 |
| Bao et al. 2010 | High | 7 | 1 | | 1 | 1 | 1 | 0 | | 1 | 1 | 1 |
| Wei et al. 2015 | Medium | 6 | 1 | | 1 | 0 | 1 | 0 | | 1 | 1 | 1 |
| Leung et al. 2008 | High | 8 | 1 | | 1 | 0 | 1 | 2 | | 1 | 1 | 1 |
| Hu et al. 2012 | High | 7 | 1 | | 1 | 1 | 1 | 0 | | 1 | 1 | 1 |
| Huang et al. 2016 | High | 8 | 1 | | 1 | 1 | 1 | 2 | | 1 | 1 | 0 |
| Zhang et al. 2016 | High | 9 | 1 | | 1 | 1 | 1 | 2 | | 1 | 1 | 1 |
| Yang et al. 2012 | Medium | 6 | 0 | | 1 | 1 | 1 | 0 | | 1 | 1 | 1 |
| Ren et al. 2016 | High | 8 | 1 | | 1 | 0 | 1 | 2 | | 1 | 1 | 1 |
| Wang et al. 2013 | High | 7 | 1 | | 1 | 1 | 1 | 0 | | 1 | 1 | 1 |
| Li et al. 2013 | High | 9 | 1 | | 1 | 1 | 1 | 2 | | 1 | 1 | 1 |
| Chen et al. 2010 | High | 7 | 1 | | 1 | 0 | 1 | 2 | | 1 | 1 | 0 |
| Zhang et al. 2015 | Medium | 5 | 1 | | 1 | 0 | 1 | 0 | | 0 | 1 | 1 |
| Zhu et al. 2010 | Medium | 6 | 1 | | 1 | 1 | 1 | 0 | | 1 | 1 | 0 |
| Liu et al. 2015 | High | 7 | 1 | | 1 | 1 | 1 | 0 | | 1 | 1 | 1 |
| Li et al. 2016 | Medium | 6 | 1 | | 1 | 1 | 1 | 0 | | 1 | 1 | 0 |
| Yang et al. 2012 | High | 7 | 1 | | 1 | 1 | 1 | 0 | | 1 | 1 | 1 |
| Kong et al. 2014 | Medium | 6 | 1 | | 1 | 1 | 1 | 0 | | 1 | 1 | 0 |
| Zhu et al. 2013 | Medium | 6 | 1 | | 1 | 1 | 1 | 0 | | 1 | 1 | 1 |
| Luo et al. 2005 | Medium | 6 | 1 | | 1 | 1 | 1 | 0 | | 1 | 1 | 0 |
| Xu et al. 2014 | Medium | 6 | 1 | | 1 | 1 | 1 | 0 | | 1 | 1 | 0 |
| Jiang et al. 2013 | Medium | 6 | 1 | | 1 | 1 | 1 | 0 | | 1 | 1 | 0 |
| Li et al. 2014 | High | 7 | 1 | | 1 | 1 | 1 | 0 | | 1 | 1 | 1 |
| Xia et al. 2012 | High | 7 | 1 | | 1 | 1 | 1 | 0 | | 1 | 1 | 1 |
| Chu et al. 2012 | High | 9 | 1 | | 1 | 1 | 1 | 2 | | 1 | 1 | 1 |
| Li et al. 2016 | Medium | 6 | 1 | | 1 | 0 | 1 | 0 | | 1 | 1 | 1 |
| Wang et al. 2011 | Medium | 6 | 1 | | 1 | 0 | 1 | 0 | | 1 | 1 | 1 |
| Liu et al. 2015 | Medium | 6 | 1 | | 1 | 0 | 1 | 0 | | 1 | 1 | 1 |
| Sun et al. 2016 | High | 7 | 1 | | 1 | 0 | 1 | 2 | | 1 | 1 | 0 |
| Tong et al. 2013 | Medium | 5 | 1 | | 1 | 0 | 1 | 0 | | 1 | 1 | 0 |
| Fan et al. 2015 | Medium | 6 | 1 | | 1 | 1 | 1 | 0 | | 1 | 1 | 0 |
| Liu et al. 2010 | Medium | 6 | 1 | | 1 | 1 | 1 | 0 | | 1 | 1 | 0 |
| Li et al. 2012 | Medium | 6 | 1 | | 1 | 1 | 1 | 0 | | 1 | 1 | 0 |
| Xue et al. 2014 | Medium | 5 | 1 | | 1 | 0 | 1 | 0 | | 1 | 1 | 0 |

**Table S4 Event and prevalence according to BMI**

| **Study (location)** | **Sample** | **N (%)** | | | | **Outcomes** | **Event n (%)** | | | |
| --- | --- | --- | --- | --- | --- | --- | --- | --- | --- | --- |
|  |  | Underweight | Normal | Overweight | Obese |  | Underweight | Normal | Overweight | Obese |
| Chen et al. 2013  (14 provinces) | 60729 | 7410(12.2) | 42241 (69.6) | 8817 (14.5) | 2261(3.7) | LBW | 538 (7.3) | 2312(5.5) | 554(6.3) | 151(6.7) |
| Liu et al. 2015  (Beijing) | 2973 | 254 (8.5) | 2152 (72.4) | 567(19.1) | | PTB | 11 (4.3) | 143 (6.6) | 69 (12.2) | |
|  |  |  |  |  |  | Macrosomia | 15 (5.9) | 247 (11.5) | 66 (11.6) | |
|  |  |  |  |  |  | SGA | 4 (1.6) | 15 (0.7) | 11 (1.9) | |
| Liu et al. 2011  (Liaoning) | 5047 | 579 (11.5) | 3200 (63.4) | 926 (18.3) | 342 (6.8) | PTB | 28 (4.8) | 250 (7.8) | 93 (10.0) | 57 (16.7) |
|  |  |  |  |  |  | SGA | 54 (9.3) | 186 (5.8) | 33 (3.6) | 21 (6.1) |
|  |  |  |  |  |  | LGA | 22 (3.8) | 231 (7.2) | 94 (10.2) | 44 (12.9) |
|  |  |  |  |  |  | Fetal distress | 24 (4.2) | 141 (4.4) | 32 (3.5) | 4 (1.2) |
| Wang et al.2015  (18 provinces) | 172206 | 23429 (13.6) | 129982 (75.5) | 15880 (9.2) | 2915 (1.7) | PTB | 1380(5.9) | 7058(5.4) | 945(5.9) | 184(6.3) |
| Wang et al.2011  (Hebei, Jiangsu, Zhejiang) | 353477 | 67606 (19.1) | 259481  (73.4) | 24756 (7.0) | 1634 (0.5) | PTB | 2492(3.69) | 9303 (3.59) | 949 (3.83) | 80 (4.9) |
| Ding et al.2015  (Anhui) | 10251 | 2365 (23.1) | 7240 (70.6) | 646 (6.3) | | PTB | 70(2.96) | 235(3.25) | 29 (4.49) | |
|  |  |  |  |  |  | Fetal distress | 151(6.38) | 497(6.86) | 38 (5.88) | |
| Zhang et al.2016  (Hubei) | 76695 | 13115(17.1) | 58442(76.2) | 4448(5.8) | 690(0.9) | SGA | 1516(11.6) | 4806 (8.2) | 315 (7.0) | 44 (6.6) |
| Yang et al. 2015  (Hubei) | 85765 | 14477(16.9) | 65536(76.4) | 5752(6.7) | | LBW | 475(3.28) | 1795(2.74) | 233(4.05) | |
|  |  |  |  |  |  | Macrosomia | 555(3.83) | 4371(6.67) | 665(11.56) | |
| Huang et al.2016  (6 provinces) | 17475 | 4335 (24.8) | 11559 (66.1) | 1347 (7.7) | 234 (1.3) | PTB | 161(3.71) | 373(3.23) | 67 (4.24) | |
| Wei et al. 2015  (Beijing) | 14451 | 2055 (14.2) | 9574 (66.3) | 2142 (14.8) | 680 (4.7) | PTB | 112(5.45) | 420(4.39) | 134(6.26) | 56(8.24) |
|  |  |  |  |  |  | Macrosomia | 78(3.80) | 723(7.55) | 239(11.16) | 84(12.35) |
| Leung et al.2008  (Hong Kong) | 29303 | 2629 (9.0) | 22041 (75.2) | 3956(13.5) | 677 (2.3) | PTB | 195(7.4) | 1392(6.3) | 277(7.0) | 67(9.9) |
|  |  |  |  |  |  | SGA | 395 (15) | 1931(8.8) | 238(6.0) | 35 (5.2) |
|  |  |  |  |  |  | LGA | 69 (2.6) | 1694(7.7) | 631(16.0) | 142 (21.0) |
|  |  |  |  |  |  | LGA | 69 (2.6) | 1694(7.7) | 631(16.0) | 142 (21.0) |
| Hu et al. 2012  (Jiangsu) | 33631 | 7271 (22.0) | 20339 (61.6) | 3210(9.7) | 2219 (6.7) | LBW | 228(3.14) | 569(2.80) | 116(3.61) | 90(4.06) |
|  |  |  |  |  |  | Macrosomia | 246(3.38) | 823(4.05) | 155(4.83) | 138(6.22) |
| Zhang et al.2016  (Hubei) | 11020 | 2578 (23.4) | 7285 (66.1) | 1157(10.5) | | PTB | 160(6.21) | 409(5.61) | 61(5.27) | |
| Yang et al. 2012  (Beijing) | 4736 | 465 (9.8) | 3549 (75.0) | 722(15.2) | | LBW | 10(2.15) | 22(0.62) | 6(0.83) | |
|  |  |  |  |  |  | Macrosomia | 13(2.80) | 246(6.93) | 100(13.85) | |
| Ren et al. 2016  (Beijing) | 13938 | 1985 (14.2) | 9219(66.2) | 2059(14.8) | 675 (4.8) | Macrosomia | 63(3.17) | 627(6.80) | 212(10.30) | 81(12.00) |
| Wang et al. 2013  (Beijing) | 16460 | 3089 (18.8) | 11478 (69.7) | 1893(11.5) | | LBW | 33(1.07) | 102(0.89) | 17(0.90) | |
|  |  |  |  |  |  | Macrosomia | 104(3.36) | 889(7.75) | 249(13.15) | |
| Li et al. 2013  (Tianjin) | 33973 | 3809 (11.2) | 21942 (64.6) | 6185(18.2) | 2037 (6.0) | PTB | 99(2.6) | 636(2.9) | 210(3.4) | 100(4.9) |
|  |  |  |  |  |  | LBW | 110(2.9) | 439(2.0) | 136(2.2) | 59(2.9) |
|  |  |  |  |  |  | Macrosomia | 152(4.0) | 1887(8.6) | 872(14.1) | 407(20.0) |
|  |  |  |  |  |  | SGA | 590(15.5) | 1975(9.0) | 427(6.9) | 116(5.7) |
|  |  |  |  |  |  | LGA | 152(4.0) | 1997(9.1) | 922(14.9) | 460(22.6) |
| Chen et al. 2010 (Liaoning) | 2586 | 408 (15.8) | 1744 (67.4) | 341(13.2) | 93 (3.6) | PTB | 20 (4.9) | 117 (6.7) | 47 (13.8) | 18 (19.4) |
|  |  |  |  |  |  | Macrosomia | 20 (4.9) | 176 (10.1) | 41 (12.0) | 11 (11.8) |
| Zhang et al.2015  (Beijing) | 4036 | 670 (16.6) | 2914 (72.2) | 452 (11.2) | | LBW | 9(1.3) | 31(1.1) | 5(1.1) | |
|  |  |  |  |  |  | Macrosomia | 26(3.9) | 236(8.1) | 61(13.5) | |
| Zhu et al. 2010  (Zhejiang) | 1160 | 140 (12.1) | 719 (62.0) | 301(25.9) | | Macrosomia | 7(5.00) | 44(6.10) | 36(11.96) | |
|  |  |  |  |  |  | Fetal distress | 7(5.00) | 26(3.60) | 10(3.30) | |
|  |  |  |  |  |  | neonatal asphyxia | 6 (4.30) | 20 (2.80) | 9 (3.00) | |
| Liu et al. 2015  (Beijing) | 2124 | 250 (11.8) | 1370 (64.5) | 388(18.2) | 116 (5.5) | PTB | 5(2.0) | 65(4.7) | 18(4.6) | 12(10.3) |
|  |  |  |  |  |  | Macrosomia | 14(5.6) | 150(10.9) | 46(11.9) | 14(12.1) |
| Li et al. 2016  (Henan) | 1456 | 291 (20.0) | 1048 (72.0) | 85 (5.8) | 32 (2.2) | PTB | 29(10.0) | 100(9.5) | 11(12.9) | 4(12.5) |
|  |  |  |  |  |  | LBW | 19(6.5) | 74(7.1) | 7(8.2) | 2(6.3) |
|  |  |  |  |  |  | Macrosomia | 9(3.1) | 73(7.0) | 9(10.6) | 6(18.8) |
|  |  |  |  |  |  | neonatal asphyxia | 8 (2.8) | 32 (3.1) | 3 (3.5) | 2 (6.3) |
| Yang et al. 2012  (Fujian) | 1044 | 157 (15.0) | 598 (57.3) | 289 (27.7) | | PTB | 12 (7.6) | 27 (4.5) | 16 (5.5) | |
|  |  |  |  |  |  | Macrosomia | 7 (4.5) | 23 (3.8) | 27 (9.3) | |
|  |  |  |  |  |  | neonatal asphyxia | 13 (8.3) | 24 (4.0) | 20 (6.9) | |
| Kong et al. 2014  (Jiangxi) | 1048 | 158 (15.1) | 600 (57.3) | 290 (27.6) | | PTB | 14 (8.9) | 27 (4.5) | 16 (5.5) | |
|  |  |  |  |  |  | LBW | 15 (9.5) | 25 (4.2) | 13 (4.5) | |
|  |  |  |  |  |  | Macrosomia | 7 (4.4) | 22 (3.7) | 28 (9.6) | |
|  |  |  |  |  |  | neonatal asphyxia | 13 (8.2) | 25 (4.2) | 22 (7.6) | |
| Zhu et al. 2013  (Zhejiang) | 4749 | 806 (17.0) | 3437 (72.4) | 443 (9.3) | 63 (1.3) | PTB | 48(5.96) | 145(4.22) | 28(6.32) | 4(6.35) |
|  |  |  |  |  |  | Macrosomia | 9(1.12) | 180(5.24) | 51(11.51) | 19(30.16) |
| Luo et al. 2005  (Guangxi) | 1120 | 271 (24.2) | 541 (48.3) | 308 (27.5) | | Macrosomia | 2(0.73) | 27(4 .99) | 51(16 .56) | |
|  |  |  |  |  |  | SGA | 35(12.92) | 13(2.40) | 7(2.27) | |
|  |  |  |  |  |  | neonatal asphyxia | 5(1 .85) | 12(2 .22) | 17(5 .52) | |
| Xu et al. 2014  (Zhejiang) | 1530 | 154 (10.1) | 1206 (78.8) | 170 (11.1) | | LBW | 34(22.08) | 34(2.82) | 2(1.18) | |
|  |  |  |  |  |  | Macrosomia | 2(1.30) | 45(2.18) | 18(10.59) | |
| Li et al. 2014  (14 provinces) | 48867 | 6424 (13.2) | 34543 (70.7) | 6514(13.3) | 1386 (2.8) | LBW | 205(3.2) | 644(1.9) | 112(1.7) | 20(1.4) |
|  |  |  |  |  |  | Macrosomia | 245(3.8) | 2185(6.3) | 772(11.9) | 243(17.5) |
|  |  |  |  |  |  | Neonatal death | 14(0.2) | 95(0.3) | 13(0.2) | 4(0.3) |
| Xia et al. 2012  (Jiangxi) | 1200 | 108 (9.0) | 813 (67.8) | 279 (23.2) | | LBW | 6(5.56) | 7(0.86) | 2(0.72) | |
|  |  |  |  |  |  | Macrosomia | 2(1.85) | 86(10.58) | 77(27.60) | |
|  |  |  |  |  |  | neonatal asphyxia | 3(2.78) | 23(2.83) | 14(5.02) | |
| Chu et al. 2012  (Anhui) | 12355 | 2834 (22.9) | 8745 (70.8) | 776 (6.3) | | LBW | 67(2.36) | 160(1.83) | 5 (0.64) | |
|  |  |  |  |  |  | Macrosomia | 171(6.03) | 937(10.71) | 144 (18.56) | |
|  |  |  |  |  |  | SGA | 135(4.76) | 284(3.25) | 18 (2.32) | |
|  |  |  |  |  |  | LGA | 473(16.69) | 2202(25.18) | 288(37.1) | |
| Li et al. 2016  (Guangdong) | 3200 | 640 (20.0) | 2208 (69.0) | 304(9.5) | 48 (1.5) | PTB | 58(9.1) | 212(9.6) | 40(13.2) | 6(12.5) |
|  |  |  |  |  |  | Macrosomia | 7(1.1) | 132(6.0) | 33(11.0) | 9(18.8) |
|  |  |  |  |  |  | neonatal asphyxia | 18(2.8) | 68(3.1) | 10(3.3) | 2(4.2) |
| Wang et al. 2011  (Anhui) | 1419 | 245 (17.3) | 1006 (70.9) | 168 (11.8) | | SGA | 33(13.5) | 62(6.2) | 11(6.5) | |
|  |  |  |  |  |  | LGA | 30(12.2) | 228(22.7) | 63(37.5) | |
| Liu et al. 2015  (Henan) | 1245 | 92 (7.4) | 736 (59.1) | 417 (33.5) | | Macrosomia | 4(4.4) | 36(4.9) | 47(11.3) | |
|  |  |  |  |  |  | Fetal distress | 3(3.3) | 16(2.2) | 23(5.5) | |
|  |  |  |  |  |  | neonatal asphyxia | 4(4.4) | 18(2.4) | 27(6.5) | |
| Sun et al. 2016  (Jiangsu) | 3943 | 540 (13.7) | 2761 (70.0) | 642(16.3) | | PTB | 20(3.7) | 78(2.8) | 33(5.1) | |
|  |  |  |  |  |  | LBW | 18(3.3) | 57(2.1) | 27(4.2) | |
|  |  |  |  |  |  | SGA | 13(2.4) | 68(2.5) | 23(3.6) | |
| Tong et al. 2013  (Nanjing) | 2409 | 445 (18.5) | 1685 (69.9) | 279 (11.6) | | PTB | 17(3.8) | 65(3.9) | 15(5.4) | |
|  |  |  |  |  |  | Macrosomia | 17(3.8) | 132(7.8) | 38(13.6) | |
| Fan et al. 2015  (Hainan) | 1743 | 422 (24.2) | 1169 (67.1) | 152(8.7) | | Macrosomia | 31(7.4) | 128(11.0) | 32(21.1) | |
|  |  |  |  |  |  | LGA | 84(19.9) | 331(28.3) | 51(33.6) | |
| Liu et al. 2010  (Liaoning) | 3741 | 540 (14.4) | 2404 (64.3) | 598 (16.0) | 199 (5.3) | PTB | 26(4.8) | 188(7.8) | 70(11.7) | 34(17.1) |
|  |  |  |  |  |  | SGA | 50(9.3) | 140(5.8) | 37(6.2) | 23(11.6) |
|  |  |  |  |  |  | LGA | 21(3.9) | 177(7.4) | 72(12.0) | 30(15.1) |
| Li et al. 2012  (Anhui) | 1628 | 271 (16.7) | 1075 (66.0) | 282 (17.3) | | PTB | 6(2.2) | 47(4.4) | 13(4.6) | |
|  |  |  |  |  |  | Macrosomia | 7(2.6) | 114(10.6) | 48(17.0) | |
|  |  |  |  |  |  | Fetal distress | 30(11.1) | 114(10.6) | 18(6.4) | |
|  |  |  |  |  |  | neonatal asphyxia | 5(1.8) | 30(2.8) | 12(4.3) | |
| Xue et al. 2014  (Beijng) | 3840 | 699 (18.2) | 2692 (70.1) | 449 (11.7) | | LBW | 3 (0.4) | 24 (0.9) | 3 (0.7) | |
|  |  |  |  |  |  | Macrosomia | 27(3.9) | 213(7.9) | 80(17.8) | |

LGA, large for gestational age; SGA, small for gestational age; LBW, low birth weight; PTB, premature birth.

**Table S5** Odds ratio (95% CI) by BMI categories and outcomes

| **Study (location)** | **Sample** | **Outcomes** | **Crude odd ratios (95% confidence interval)** | | |  | **Adjusted odd ratios (95% confidence interval)** | | |
| --- | --- | --- | --- | --- | --- | --- | --- | --- | --- |
|  |  |  | **Underweight** | **Overweight** | **Obese** |  | **Underweight** | **Overweight** | **Obese** |
| Chen et al. 2013  (14 provinces) | 60729 | LBW | 1.637 (1.42, 1.89) | 0.840(0.72,0.98) | 0.620(0.47,0.83) |  | – | – | – |
| Liu et al. 2015  (Beijing) | 2973 | PTB | – | – | |  | 0.69 (0.32, 1.13) | 2.51 (1.83, 3.45) | |
|  |  | Macrosomia | – | – | |  | 0.53 (0.30, 0.92) | 0.79 (0.58, 1.08) | |
|  |  | SGA | – | – | |  | 1.97 (0.63, 6.11) | 2.81 (1.21, 6.54) | |
| Lei et al. 2016  (Guangdong) | 5535 | PTB | – | – | – |  | 1.00(0.77, 1.30) | 1.54(1.14, 2.08) | 1.42(1.02, 1.97) |
|  |  | SGA | – | – | – |  | 1.62(1.13, 2.34) | 0.71(0.38, 1.34) | 0.69(0.34, 1.37) |
|  |  | LGA | – | – | – |  | 0.65(0.52, 0.82) | 1.32(1.02, 1.71) | 2.35(1.81, 3.04) |
|  |  | Neonatal asphyxia | – | – | – |  | 1.60(0.80, 3.19) | 0.99(0.34, 2.85) | 2.38(1.06, 5.30) |
| Liu et al. 2011  (Liaoning) | 5047 | PTB | – | – | – |  | 0.6 (0.3, 1.1) | 1.3 (0.5, 2.0) | 2.3 (0.9, 4.9) |
|  |  | SGA | – | – | – |  | 1.7 (1.1, 2.6) | 0.6 (0.4, 0.9) | 1.1 (0.7, 1.7) |
|  |  | LGA | – | – | – |  | 0.5 (0.3, 1.0) | 1.5 (1.0, 2.1) | 1.9 (1.2, 3.1) |
|  |  | Fetal distress | – | – | – |  | 0.9 (0.5, 1.7) | 0.8 (0.4, 1.3) | 0.3 (0.1, 1.1) |
| Du et al. 2017  (Zhejiang) | 3772 | Macrosomia | – | – | – |  | 0.38(0.21, 0.68) | 2.90(1.99, 4.23) | 6.3(3.42, 11.47) |
|  |  | SGA | – | – | – |  | 1.86(1.39, 2.50) | 0.43(0.25, 0.74) | 0.54(0.21, 1.40) |
|  |  | LGA | – | – | – |  | 0.41(0.27, 0.63) | 2.23(1.66, 2.99) | 3.99(2.41, 6.60) |
| Wang et al.2015  (18 provinces) | 172206 | PTB | 1.09 (1.03,1.16) | 1.10 (1.03,1.18) | 1.17 (1.01,1.36) |  | – | – | – |
| Pan et al. 2016  (31 provinces) | 536098 | PTB | 1.16 (1.08, 1.24) | 1.03 (0.94, 1.12) | 1.21 (1.02, 1.44) |  | 1.16 (1.08, 1.25) | 1.01(0.92, 1.10) | 1.18 (0.99, 1.4) |
|  |  | LBW | 1.64 (1.46, 1.84) | 1.22 (1.05, 1.42) | 1.61 (1.22, 2.14) |  | 1.57 (1.4, 1.77) | 1.22 (1.05, 1.42) | 1.60 (1.2, 2.12) |
| Li et al. 2015  (14 provinces) | 48867 | SGA | – | – | – |  | 1.66 (1.44, 1.91) | 0.73 (0.59, 0.91) | 1.09 (0.67, 1.77) |
|  |  | LGA | – | – | – |  | 0.54 (0.47, 0.62) | 2.55 (2.32, 2.80) | 3.95 (3.20, 4.87) |
| Wang et al.2011  (Hebei, Jiangsu, Zhejiang) | 353477 | PTB | – | – | – |  | 1.05 (1.00, 1.10) | 1.08 (1.01, 1.15) | 1.43 (1.14,1.79) |
| Ding et al.2015  (Anhui) | 10251 | PTB | 0.8 (0.5, 1.2) | 0.7 (0.3, 1.6) | |  | 0.8 (0.5, 1.2) | 0.8 (0.4, 1.7) | |
|  |  | Fetal distress | 0.9 (0.7, 1.1) | 0.9 (0.6, 1.2) | |  | 1.0 (0.8, 1.2) | 0.9 (0.6, 1.2) | |
| Zhang et al.2016  (Hubei) | 76695 | SGA | 1.41 (1.33, 1.49) | 0.85 (0.77, 0.95) | 0.80 (0.60, 1.06) |  | 1.40 (1.32, 1.48) | 0.87 (0.78, 0.97) | 0.82 (0.62, 1.10) |
| Huang et al.2016  (6 provinces) | 17475 | PTB | 1.16 (0.96, 2.00) | 1.30 (0.98, 1.73) | 1.48 (0.80, 2.74) |  | – | – | – |
| Bao et al. 2010  (Harbin) | 13711 | Macrosomia | – | – | – |  | – | 2.17 (1.60, 2.94) | 5.81 (4.26, 7.92) |
| Wei et al. 2015  (Beijing) | 14451 | PTB | 1.32 (1.07, 1.64) | 1.38 (1.13, 1.69) | 1.90 (1.42, 2.55) |  | – | – | – |
|  |  | Macrosomia | 0.48 (0.38, 0.61) | 1.55 (1.33, 1.81) | 1.74 (1.36, 2.21) |  | – | – | – |
| Huang et al.2016  (Anhui) | 3081 | SGA | – | – | – |  | 1.67 (1.25, 2.23) | 0.91 (0.56, 1.47) | |
|  |  | LGA | – | – | – |  | 0.61 (0.44, 0.83) | 1.50 (1.11, 2.04) | |
| Zhang et al.2016  (Hubei) | 11020 | PTB | – | – | – |  | 1.07 (0.88, 1.31) | 0.87 (0.65, 1.16) | |
| Ren et al. 2016  (Beijing) | 13938 | Macrosomia | 0.45 (0.34, 0.58) | 1.57 (1.34, 1.85) | 1.87 (1.46, 2.39) |  | 0.42 (0.32, 0.56) | 1.70 (1.43, 2.02) | 2.39 (1.83, 3.13) |
| Li et al. 2013  (Tianjin) | 33973 | PTB | – | – | – |  | 0.93 (0.75, 1.15) | 1.15 (0.98, 1.35) | 1.70 (1.36, 2.11) |
|  |  | LBW | – | – | – |  | 1.73 (1.35, 2.22) | 0.92 (0.72, 1.16) | 0.96 (0.68, 1.35) |
|  |  | Macrosomia | – | – | – |  | 0.45 (0.38, 0.53) | 1.76 (1.62, 1.93) | 2.86 (2.53, 3.23) |
|  |  | SGA | – | – | – |  | 1.84 (1.66, 2.03) | 0.75 (0.67, 0.83) | 0.61 (0.50, 0.74) |
|  |  | LGA | – | – | – |  | 0.42 (0.36, 0.50) | 1.73 (1.59, 1.88) | 2.80 (2.49, 3.15) |
| Chen et al. 2010  (Liaoning) | 2586 | PTB | – | – | – |  | 0.85 (0.51, 1.40) | 1.57 (1.06, 2.34) | 1.45 (0.78, 2.70) |
|  |  | Macrosomia | – | – | – |  | 0.48 (0.30, 0.77) | 1.27 (0.87, 1.86) | 1.21 (0.61, 2.41) |
| Jiang et al. 2013  (Henan) | 2241 | PTB | – | 1.18 (0.41, 2.12) | 2.35 (0.89, 3.98) |  | – | – | – |
|  |  | SGA | – | 0.76 (0.45, 0.99) | 1.02 (0.67, 1.76) |  | – | – | – |
|  |  | LGA | – | 1.56 (1.01, 2.31) | 1.71 (1.07, 3.20) |  | – | – | – |
|  |  | Fetal distress | – | 0.67 (0.34, 1.39) | 0.31 (0.08, 1.35) |  | – | – | – |
| Chu et al. 2012  (Anhui) | 12355 | LBW | – | – | |  | 1.22 (0.91, 1.63) | 0.39 (0.16, 0.95) | |
|  |  | Macrosomia | – | – | |  | 0.54 (0.46, 0.64) | 1.87 (1.54, 2.27) | |
|  |  | SGA | – | – | |  | 1.32 (1.07, 1.63) | 0.86 (0.53, 1.40) | |
|  |  | LGA | – | – | |  | 0.67 (0.54, 0.68) | 1.76 (1.50, 2.06) | |
| Fan et al. 2015  (Hainan) | 1743 | Macrosomia | – | – | |  | 0.74 (0.3, 0.8) | 2.09 (1.2, 2.9) | |
|  |  | LGA | – | – | |  | 0.60 (0.3, 0.9) | 1.13 (0.8, 1.6) | |
| Liu et al. 2010  (Liaoning) | 3741 | PTB | – | – | – |  | 0.60 (0.34, 1.04) | 1.36 (0.93, 1.98) | 1.50 (0.85, 2.65) |
|  |  | SGA | – | – | – |  | 1.67 (1.07, 2.59) | 0.60 (0.45, 0.97) | 1.19 (0.59, 2.43) |
|  |  | LGA | – | – | – |  | 0.51 (0.28, 0.93) | 1.74 (1.21, 2.49) | 2.24 (1.34, 3.74) |
