# Supplementary material for: Maternal body mass index and risk of neonatal adverse outcomes in China: a systematic review and meta-analysis
Source: BMC Pregnancy Childbirth. 2019 Mar 29;19:105. doi: 10.1186/s12884-019-2249-z (PMC6440121; doi:10.1186/s12884-019-2249-z)
Supplement: Supplementary file 3 — Forest plot and funnel plot of the association between maternal BMI and neonatal adverse outcomes. (DOC 167 kb) [file 12884_2019_2249_MOESM3_ESM.doc]

**Additional file 3 Forest plot and funnel plot of the association between maternal BMI and neonatal adverse outcomes**

Figure S1 Forest plot of the association between maternal BMI and LBW

(a) underweight vs. normal weight

(b) overweight/obesity vs. normal weight

Figure S2 Forest plot of the association between maternal BMI and SGA

(a) underweight vs. normal weight

(b) overweight/obesity vs. normal weight

**Figure S3** Forest plot of the association between maternal BMI and macrosomia

(a) underweight vs. normal weight

(b) overweight/obesity vs. normal weight

Figure S4 Forest plot of the association between maternal BMI and LGA

(a) underweight vs. normal weight

(b) overweight/obesity vs. normal weight

**Figure S5** Forest plot of the association between maternal BMI and PTB

(a) underweight vs. normal weight

(b) overweight/obesity vs. normal weight

**Figure S5** Forest plot of the association between maternal BMI and PTB

Figure S6 Forest plot of the association between maternal BMI and Fetal Distress

(a) underweight vs. normal weight

(b) overweight/obesity vs. normal weight

**Figure S7** Forest plot of the association between maternal BMI and Neonatal Asphyxia

(a) underweight vs. normal weight

(b) overweight/obesity vs. normal weight

Figure S8 Funnel plot of the association between maternal BMI and LBW

(a) underweight vs. normal weight (Egger test P = 0.145)

(b) overweight/obesity vs. normal weight (Egger test P = 0.329)

**Figure S9** Funnel plot of the association between maternal BMI and SGA

(a) underweight vs. normal weight (Egger test P = 0.220)

(b) overweight/obesity vs. normal weight (Egger test P = 0.070)

**Figure S10** Funnel plot of the association between maternal BMI and macrosomia

(a) underweight vs. normal weight (Egger test P = 0.064)

(b) overweight/obesity vs. normal weight (Egger test P = 0.696)

**Figure S11** Funnel plot of the association between maternal BMI and LGA

(a) underweight vs. normal weight (Egger test P = 0.475)

(b) overweight/obesity vs. normal weight (Egger test P = 0.147)

**Figure S12** Funnel plot of the association between maternal BMI and PTB

(a) underweight vs. normal weight (Egger test P = 0.399)

(b) overweight/obesity vs. normal weight (Egger test P = 0.003)

**Figure S13** Funnel plot of the association between maternal BMI and Fetal Distress

(a) underweight vs. normal weight (Egger test P = 0.031)

(b) overweight/obesity vs. normal weight (Egger test P = 0.408)

**Figure S14** Funnel plot of the association between maternal BMI and Neonatal Asphyxia

(a) underweight vs. normal weight (Egger test P = 0.910)

(b) overweight/obesity vs. normal weight (Egger test P = 0.483)
